# Assessment of Galileo High Accuracy Service (HAS) test signals and preliminary positioning performance

**DOI:** 10.1007/s10291-023-01410-y

**Published:** 2023-02-15

**Authors:** Nacer Naciri, Ding Yi, Sunil Bisnath, F. Javier de Blas, Roberto Capua

**Affiliations:** 1 Department of Earth and Space Science and Engineering, York University, Toronto, ON M3J 1P3 Canada; 2 European Union Agency for the Space Programme, Prague, Czech Republic; 3 SOGEI S.P.A., Rome, Italy

**Keywords:** Galileo, High accuracy service, Precise point positioning

## Abstract

The Galileo High Accuracy Service (HAS) is a GNSS augmentation that provides precise satellite corrections to users worldwide for free directly through Galileo’s E6 signal. The HAS service provides free PPP corrections from the Galileo constellation and the Internet, with targeted real-time 95% positioning performance of better than 20 cm horizontal and 40 cm vertical error after 5 min of convergence time globally and shorter in Europe. The HAS initial service, under validation at the time of writing, provides these capabilities with a reduced performance (based on the current Galileo stations network). Live HAS test signals broadcasted from the Galileo satellites during summer 2022 have been decoded and analyzed. Corrections include Galileo and GPS orbit, clock, and code bias corrections, with SISRE of 10.6 cm and 11.8 cm for Galileo and GPS, respectively. Code bias corrections showed good performance as well, with rms of 0.28 ns, 0.26 ns, and 0.22 ns for Galileo C1C–C5Q, C1C–C7Q, and C1C–C6C, respectively, and 0.20 ns for GPS C1C–C2L. Float PPP positioning performance results show that the combined Galileo and GPS solution can already achieve the HAS full service accuracy performance target and is close in terms of convergence time, with 95% rms of 13.1 cm and 18.6 cm horizontally and vertically, respectively, in kinematic mode, and with a 95% convergence time of 7.5 min. The latter is expected to be improved with the inclusion of satellite phase bias and local atmospheric corrections. With these early Galileo HAS test signals, this preliminary analysis indicates that the HAS full service targets are attainable. Finally, a correction latency analysis is performed, showing that even with latency of up to 60 s, positioning can remain within the targeted HAS accuracy performance.

## Introduction

Galileo Improved Services for Cadastral Augmentation Development On-field Validation—GISCAD-OV (GISCAD-OV 2022; Naciri et al. 2022) is a cadastral surveying project resulting from a consortium of 14 members, with the purpose to “design, develop and validate an innovative and cost-effective High Accuracy Service for Cadastral Surveying applications, based on GPS + Galileo High Accuracy Service (HAS) and Precise Point Positioning-Ambiguity Resolution (PPP-AR) quick convergence advanced techniques” (GISCAD-OV 2022). For geodetic surveys, the Real-Time Kinematic (RTK) and Network RTK (NRTK) techniques have been widely accepted as reliable and mature technologies that can achieve centimeter-level positioning accuracy in seconds (Gao et al. 1997; Jensen and Cannon 2000; Townsend et al. 1999). Users receive Observation Space Representation (OSR) messages, which correspond to range corrections from a single reference station or a local reference network to eliminate/reduce most of the GNSS errors. Alternatively, Precise Point Positioning (PPP) and PPP-RTK have proven to achieve similar levels of positioning accuracy with a State Space Representation (SSR) of corrections, e.g., precise satellite orbit and clock products, and a standalone GNSS receiver (Bisnath and Gao 2009; Geng et al. 2011; Malys and Jensen 1990; Teunissen et al. 2010; Zumberge et al. 1997), and these corrections are either broadcast through the Internet, or transmitted by commercial satellites.

In addition to the broadcasting of SSR corrections over the Internet and commercial satellites, a recent trend has seen the emergence of the broadcasting of SSR corrections through GNSS constellations directly. The Centimeter-Level Augmentation Service (CLAS) in the Japanese Quasi-Zenith Satellite System (QZSS) was the first satellite centimeter-level augmentation service to become operational (Miya et al. 2016). The service provides QZSS and GPS PPP-RTK corrections over areas with QZSS coverage around Japan. The Chinese BeiDou has a similar regional PPP service called PPP-B2b, where multi-GNSS (only BeiDou and GPS at the moment) PPP corrections are broadcast to users in China and its surrounding areas through BeiDou’s GEO satellites over the B2b signal (Liu et al. 2020). Both QZSS CLAS and BeiDou PPP-B2b provide decimeter-level positioning (Hao et al. 2020; Tao et al. 2021; Xu et al. 2021).

Similarly to these regional services, the European Union is developing the Galileo High Accuracy Service (HAS), which is expected to be the pioneering GNSS in providing PPP orbit, clock, and code and phase bias corrections worldwide for free through its E6 signal (EUSPA 2020). The full service target performance is to allow for 95% horizontal and vertical positioning accuracy of better than 20 cm and 40 cm, respectively, within 5 min worldwide, and shorter over Europe, with 99% availability. Galileo HAS is designed to support the GPS and Galileo constellations on the L1/L5/L2C and E1/E5b/E5a/E6/E5AltBOC signals, respectively. The Galileo High Accuracy Initial Service (SL1 provision only based on the current Galileo stations’ network) has been announced to be operational on January 24th, 2023 (EUSPA 2023) by the EU Agency for the Space Programme (EUSPA), responsible for the Galileo service’s provision. The service’s maturity can benefit not only geodetic applications such as cadastral surveying, offshore exploration, and civil engineering, but also emerging markets such as Intelligent Transportation Systems (ITS), unmanned vehicles and drones, and augmented reality (EUSPA 2020).

As part of the HAS testing, Fernandez-Hernandez et al. (2022) analyzed early versions of the test signals, where simulated closed-loop tests were performed using data from September 2020, as well as signal-in-space tests using data from May 2021. The results showed that the SL1 target specifications after convergence could be met under nominal conditions and with good visibility conditions, with the 95% Signal-In-Space Ranging Error (SISRE) being 9.5 cm and 16 cm for Galileo and GPS, respectively. Hauschild et al. (2022) also made use of early HAS test signals from September 2021 and focused on precise orbit determination of satellites in low Earth orbit (LEO).

In this work, an in-depth analysis of the HAS corrections and their PPP performance from a campaign in summer 2022 is performed, where Galileo and GPS satellite orbit, clock, and code bias corrections were broadcast. The main goals of this paper are to comprehensively analyze the broadcasted test HAS corrections’ quality by assessing (1) correction availability, (2) satellite orbit and clock corrections and SISRE, (3) satellite code bias corrections, and (4) float PPP solutions using worldwide stations. The PPP performance is compared to the HAS Full Service expected performance (EUSPA 2020). It should be noted that the test infrastructure that supported Phase 0 activities is not fully representative of the future HA initial service infrastructure.

First, details about HAS and its application method on the user side are provided, followed by an analysis of the corrections availability. The satellite products are analyzed by comparing the satellite orbits and clocks to reference values from the Center for Orbit Determination in Europe (CODE), as well as analyzing the SISRE; in addition to analyzing the code bias corrections to reference values from the German Aerospace Center (DLR). The PPP performance is assessed next at the 95th and 67th percentiles in both static and kinematic modes and compared to the targeted HAS full service performance (EUSPA 2020). A simulation of the effect of HAS correction latency on PPP solutions is also performed. The paper ends with conclusions and future work.

### Galileo HAS description

The Galileo HAS corrections are transmitted on the E6B signal (1278.75 MHz) within the Galileo C/NAV navigation message. The corrections are transmitted in a format similar to Compact-SSR (CSSR), with the main differences being in the flexibility of message content and update/validity intervals. Indeed, the format used for HAS allows for, i.e., the transmission of orbits and clocks in the same message or at different rates, and a faster clock refresh rate for satellites with less stable clocks.

In this work, the decoding of HAS messages was performed by NovAtel, one of the members of the GISCAD-OV project. Several NovAtel PwrPak 7 receivers were modified and updated to decode E6B signals in real-time. One receiver is permanently installed within the GISCAD-OV Control Centre in Rome to continuously gather and log HAS messages. The decoded messages are output in a proprietary ASCII format, which can be used to process observation data with HAS corrections.

The Galileo High Accuracy Service is intended to be deployed in two phases: HAS Initial Service (delivering HAS Service Level 1—SL1 based on the current Galileo stations network) and HAS Full Service (delivering SL1 and SL2 with their target performance) (EUSPA 2020). The HAS SL1 includes the worldwide transmission, via the Signal-in-Space (SiS) and the Internet, of Galileo and GPS satellite orbits, clocks, and code and phase biases, with targeted 95% horizontal and vertical accuracies of 20 cm and 40 cm, respectively. The HAS SiS Interface Control Document (ICD) was published in May 2022, confirming the structure and contents of the HAS Signal (European Union 2022). SL2 is expected to include atmospheric (troposphere and ionosphere) corrections over the European Coverage Area (ECA), which is expected to improve the targeted convergence time from less than 300 s in SL1 to less than 100 s in SL2 in Europe. Galileo HAS has been in phase 0 in 2021 and 2022.

The HAS corrections are generated using the current Galileo Ground Stations (GSS) network of 14 stations (Fernandez-Hernandez et al. 2022), part of the existing Galileo infrastructure (EUSPA 2022). Once the corrections are generated, they are transmitted back to the Galileo satellites through the 5 up-link stations that are already used to update navigation messages and maintain the constellation in the Galileo infrastructure. The GSS network is enabling the input data for the HAS Initial Service as well, though more ground stations are expected to be added to achieve the HAS Full Service and related target performance to enable SL1 and SL2.

Considering the scope of the current HAS testing phase as confirmed by EUSPA, the corrections in the HAS SiS and related performance may not be fully representative of the HAS Initial Service and differ from one test session to the next. In this study, results from a test broadcast session in summer 2022 are analyzed, where the transmitted corrections consisted of Galileo and GPS satellite orbits, clocks, and code biases. Phase biases were set as unavailable, though the messages were included in the structure for functional validation.

### Galileo HAS Signal-in-Space

Given the real-time transmission of the corrections and the need to reduce the size of the broadcast messages, the HAS corrections are transmitted in a State Space Representation. The transmitted corrections should be used, along with the broadcast satellite orbits and clocks, to recover the precise satellite orbits and clocks. The transmitted orbital corrections are in the radial, along-track, and cross-track (RAC) directions and refer to the I/NAV and LNAV ionosphere-free antenna phase centers for Galileo and GPS, respectively (European Union 2022). The orbit corrections need to be converted to an Earth-Centered Earth-Fixed (ECEF) frame before being added to the broadcast orbits. The conversion from RAC to ECEF frames can be found in European Union (2022). Once the ECEF corrections are recovered, the precise ECEF satellite orbits can be computed by simply adding the ECEF corrections to the broadcast ECEF orbits. The precise satellite orbits will refer to the ionosphere-free antenna phase center in the Galileo Terrestrial Reference Frame (GTRF). For triple- or quadruple-frequency user processing in uncombined mode (without forming linear combinations of measurements), it is necessary to ensure that the satellite orbits on each frequency refer to that frequency’s antenna phase center using satellite antenna corrections.

A similar strategy is to be used for the satellite clocks, as the HAS corrections must be added to the broadcast satellite clocks. The reference time for Galileo HAS is the Galileo System Time (GST), and the corrections are to be added to the ionosphere-free broadcast satellite clock. One must ensure that the relativistic effect is corrected for as well when adding the HAS clock corrections to the broadcast clocks. During the processing, the user must apply code biases to offset between the frequency bias of each signal and the ionosphere-free combination of the biases that are present in the satellite clock. In general, the Observable-specific Signal Biases (OSBs) are to be applied to the pseudorange measurements on the corresponding frequencies. The same principle holds for phase biases, though they are not part of this study, as they have not been transmitted as part of the testing campaigns.

Given that navigation messages are updated up to every 10 min for Galileo and every 2 h for GPS, the HAS corrections have to be matched to their corresponding navigation message through an Issue Of Data (IOD) identifier, which is broadcasted along with the HAS corrections. The IOD should be used to find the corresponding IODnav and IODE (IOD Ephemeris)/IODC (IOD Clock) navigation messages for Galileo and GPS, respectively. The IOD range is 0–1023 for Galileo and 0–255 for GPS.

The design of the HAS messages is such that the corrections are constrained to be within certain values. Table 1 shows the range of each correction. Phase bias and atmospheric corrections are omitted, as they have not been transmitted as part of the testing campaigns. In addition to being constrained to certain ranges, the corrections can take certain values to indicate the status of the correction. For instance, all corrections have a specific value each to indicate that the correction is not available, while the satellite clocks are used to indicate to the user that the satellite’s corrections are not to be used. Such indicators provide some indication to the user on the health of a satellite’s corrections and allow the rejection of unusable corrections.

**Table 1:** Range and specific indicators of the HAS satellite orbit, clock, and code bias corrections

Correction type	Range (m)	Indicators	Validity interval (s)	Update interval (s)
Orbits—radial	± 10.2375	− 10.24: data not available	300	50
Orbits—along-track	± 16.376	− 16.384: data not available	300	50
Orbits—cross-track	± 16.376	− 16.384: data not available	300	50
Delta clocks	± 40.95	− 40.96: data not available 40.95: satellite should not be used	60	10
Code biases	± 20.46	− 20.48: data not available	3600	50

Validity and update interval values are specific to test campaigns. The indicators are specific to the NovAtel decoder format, and binary values for HAS can be found in (European Union 2022)

### Availability of HAS corrections

**Fig. 1:**
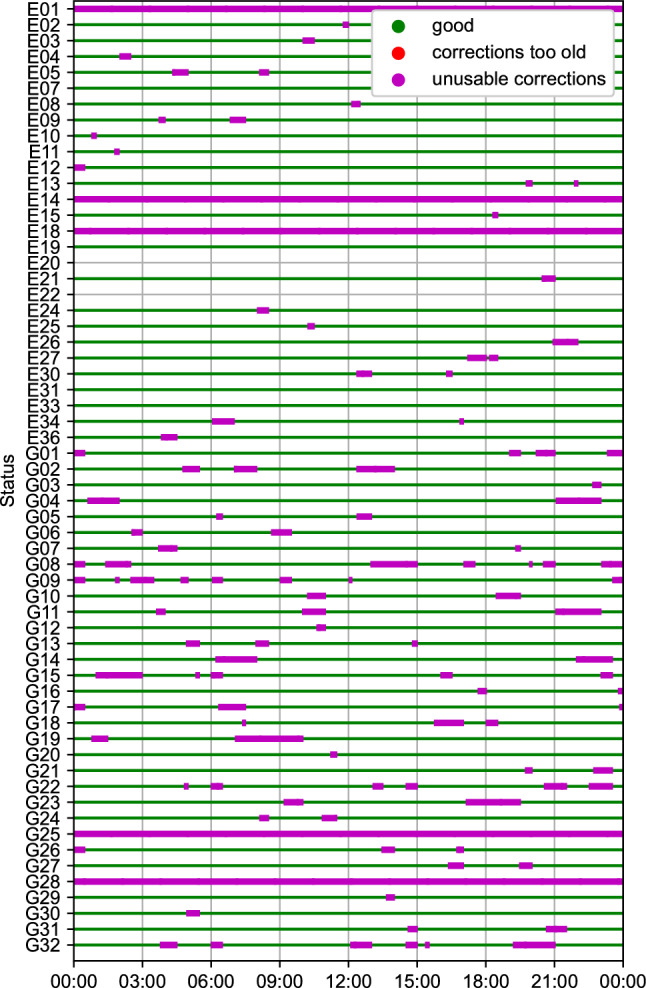
Availability of HAS orbit and clock corrections on day 247 of year 2022 for Galileo and GPS satellites

The following section analyzes the availability of HAS corrections during the campaign that took place during summer 2022, with a focus between days 242 and 248 of year 2022. The campaign consisted of Galileo and GPS orbits, clocks, and code biases. The availability analysis only involves orbits and clocks, as code biases were always available during the campaign given their longer validity intervals. As an indicator of the availability of corrections for both constellations, Fig. 1 shows the status for each satellite on day 247 of year 2022. Three main statuses are identified:

- Good: the corrections are good to be processed by the user,
- Corrections too old: the latest corrections available are past their validity interval,
- Unusable corrections: the corrections contain one of the indicators in Table 1 to indicate unavailable corrections or corrections to not be used.

Figure 1 is generated by computing the availability every 30 s over the course of the day. As the figure shows, the corrections are available throughout the day, with brief periods without corrections for certain satellites, which are more frequent for GPS than for Galileo. Satellites E20 and E22 are missing availability status, due to E22 being set as “not usable” and E20 being set as “not available” by the Galileo constellation. Additionally, some satellites have their HAS corrections set as unusable for the duration of the day (E01, E14, E18, G25, and G28), due to the status of the satellites by their constellation, e.g., E01 has been set as “not available” since DOY 243, 2022 by the Galileo constellation, while E14 and E18 are set as “not usable” satellites. PRN 28 is not attributed to any GPS satellite, and G25 has been set as “unusable” by the GPS constellation since DOY 231, 2022. In terms of the intermittent outages, they are due to the corrections being set as unusable in the decoded HAS messages. It is apparent from Fig. 1 that the outages are more frequent for GPS than Galileo. The overall availability appears to be relatively reasonable, as the intermittent outages for most satellites appear at different times of the day for each satellite. Therefore, a PPP solution would not be reinitialized, as most satellites would be processed continuously.

**Fig. 2:**
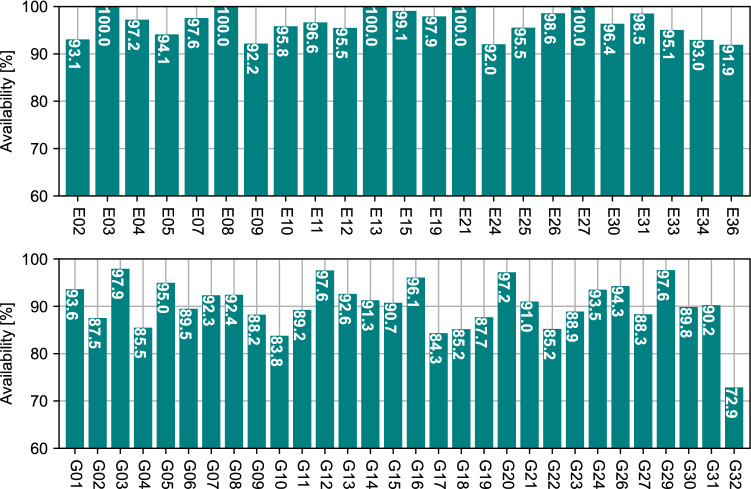
Percentage of time with available HAS orbit and clock corrections per Galileo (top) and GPS (bottom) satellite between days 242 and 248 of year 2022

To quantify correction availability, Fig. 2 details the availability per satellite over seven days between days 242 and 248 of year 2022. The figure shows that all Galileo satellites with available corrections have corrections available at least 90% of the time, with some satellites having corrections available 100% of the time. GPS satellites have a relatively lower average availability of 90.7%, due to the intermittent outages shown in Fig. 1, as compared to 96.5% for Galileo.

## Assessment of satellite orbit and clock corrections

The HAS orbit and clock corrections are compared to final orbit and clock products from the Center for Orbit Determination in Europe (CODE). Given that the products from CODE are final products, which are the highest quality of products, they can be used as references to compare against HAS products. The analysis is performed for the summer 2022 test broadcast, which contained both Galileo and GPS corrections. The analysis consists of direct comparison of the products, as well as analysis of the Signal-In-Space Ranging Error (SISRE).

The direct comparison consists of analyzing the difference between orbit and clock products in the RAC frame. To do so, the difference between both sets of clocks (HAS and CODE) is taken, and the mean value per constellation removed. The reason behind removing the mean value is that clock products tend to have common variations between satellites from the same constellation, which would not impact the user solution, as the common variations would be absorbed by the receiver clock. These common variations would be different between analysis centers, depending on the models used in the generation of the satellite products. Additionally, one has to keep in mind that HAS clocks refer to the ionosphere-free combination of the LNAV signals (L1 C/A and L2P (Y), or C1C and C2P in RINEX3 convention) for GPS and I/NAV signals (E1/E5b, or C1C and C7Q in RINEX3 convention) for Galileo (European Union 2022), while the CODE final products refer to the ionosphere-free combination of C1W and C2W (in RINEX3 convention) signals for GPS and C1C and C5Q for Galileo (Villiger et al. 2019). Consequently, alignment between these different conventions is performed using satellite biases to refer both sets of clocks to the same combinations of signals. When it comes to orbit comparison, the ECEF coordinates from both HAS and CODE are differenced, and the conversion from ECEF to RAC is performed. Satellite antenna corrections are applied to align both sets of orbits, as the HAS orbits refer to the ionosphere-free antenna phase center, while CODE orbits refer to the center of mass.

While the direct comparison of products is a good indicator of the quality of the products, it does not necessarily reflect how such products would affect user performance. The SISRE is a closer indicator, as it takes into account, for example, the projection on the line-of-sight of the different orbit components. The SISRE depends on the user location; though it is possible to compute a global SISRE that represents the average SISRE to be expected when using the products (Montenbruck et al. 2015). In this work, two SISRE are considered: one *orbit SISRE* that only considers orbit errors, while the *total SISRE* considers the clock errors as well. Both SISRE formulations used in this work can be found in Montenbruck et al. (2015).

**Fig. 3:**
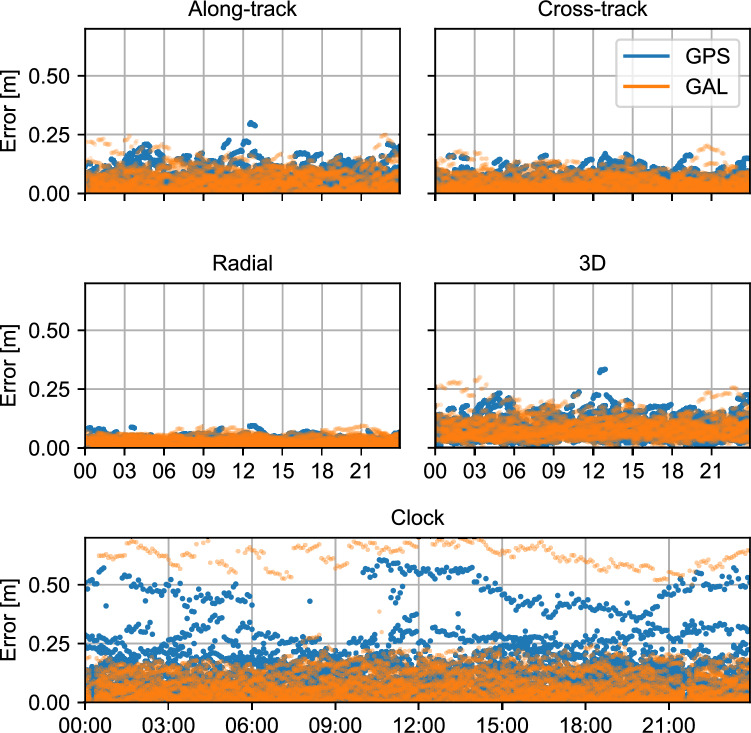
Along-track (top left), cross-track (top right), radial (center left), and 3D (center right) orbital errors and clock (bottom) errors of Galileo and GPS HAS corrections relative to final CODE orbits and clocks on day 247 of year 2022

Figure 3 highlights the orbit errors in the RAC frame, as well as the 3D combination, in addition to the clock errors for day 247 of year 2022. The mean per constellation in the clock errors is removed to eliminate common biases between HAS and CODE. The rms error for the individual components of each constellation is summarized in Table 2. In terms of orbit errors, the rms of the 3D errors is 9.0 cm and 8.6 cm for Galileo and GPS, respectively. These rms values are mostly due to the along- and cross-track components, with the radial component being 3.2 cm and 2.4 cm for Galileo and GPS, respectively. Consequently, the higher along-track rms and cross-track rms are expected to have limited impact on the user performance, as the components will be projected to the line-of-sight between the satellite and the user. Similar to the radial component, the clock component has a direct impact on user performance. The rms of the clock errors are 15.5 cm (0.52 ns) for Galileo and 14.5 cm (0.48 ns) for GPS, showing comparable rms between Galileo and GPS clocks. However, as can be seen in Fig. 3, two satellites have relatively larger clock errors: satellite G19 with a clock error around 0.4/0.5 m and satellite E12 with a clock error of around 0.6/0.7 m. The reason behind these two larger clock errors is still unknown and can be associated with the test nature of the HAS broadcast. Removing these two satellites from the computation leads to clock errors of 8.8 cm (0.29 ns) and 11.6 cm (0.39 ns) for Galileo and GPS, respectively.

**Table 2:** rms of the radial, along-track, cross-track, 3D orbit, and clock errors of HAS relative to CODE final products on day 247 of year 2022

	Radial	Along-track	Cross-track	3D	Clock	Clock excluding G19 and E12
Galileo	3.2	6.6	5.3	9.0	15.5	8.8
GPS	2.4	6.4	5.3	8.6	14.5	11.6

The rms is computed taking into account all satellites from each constellation. Values are in cm

The comparison with CODE products yields comparable results to those shown by Hauschild et al. (2022) and Fernandez-Hernandez et al. (2022), though the broadcast period and the reference products are different. BeiDou’s PPP-B2b service follows a similar principle as Galileo HAS, in the sense that PPP corrections are being broadcast from the BeiDou satellites directly. PPP-B2b consists of PPP corrections for BeiDou-3 and GPS. Comparing the values in Table 2 with already published results for PPP-B2b, it appears that GPS (the common constellation) orbit corrections from Galileo HAS have less error than in PPP-B2b, though the latter has more accurate clocks (Nie et al. 2021; Tao et al. 2021).

**Fig. 4:**
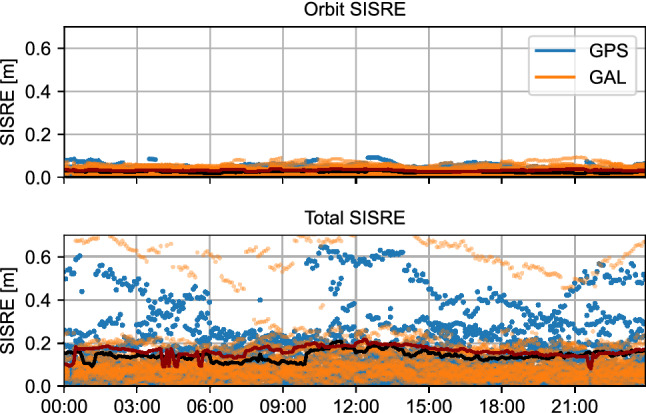
Orbital SISRE (top) and total SISRE (bottom) of Galileo and GPS HAS corrections relative to final CODE orbits and clocks on day 247 of year 2022. The horizontal dark red lines represent the Galileo rms, while the black lines represent the GPS rms. Satellites G19 and E12 are excluded from the rms computation

A SISRE analysis is performed in Fig. 4, where both the SISRE considering only orbit corrections and that considering both orbit and clock corrections are analyzed. The results behave as expected given the results in Fig. 3: both Galileo and GPS SISRE perform reasonably well, except for G19 and E12, for which the higher clock errors lead to higher total SISRE. The 24-h rms for Galileo are 3.1 cm and 10.8 cm for the orbit SISRE and total SISRE, respectively, excluding E12. In contrast, the GPS rms is 2.4 cm and 11.6 cm for the orbit SISRE and total SISRE, respectively, excluding G19. These values show comparable quality between Galileo and GPS. Comparable values were obtained by Hauschild et al. (2022) for the Galileo constellation for the September 2021 test broadcast, though GPS SISRE was higher for that campaign compared to this study of the summer 2022 test broadcast. Similar conclusions are made when comparing to results by Fernandez-Hernandez et al. (2022), which focused on the September 2020 test broadcast. Additionally, for general comparison, these GPS SISRE values appear to be lower than the PPP-B2b corrections by Tao et al. (2021), which showed larger clock variations for GPS. Compared to SISRE from Centre National d'Etudes Spatiales (CNES) products in the same paper, the HAS GPS orbit SISRE and total SISRE are comparable.

**Fig. 5:**
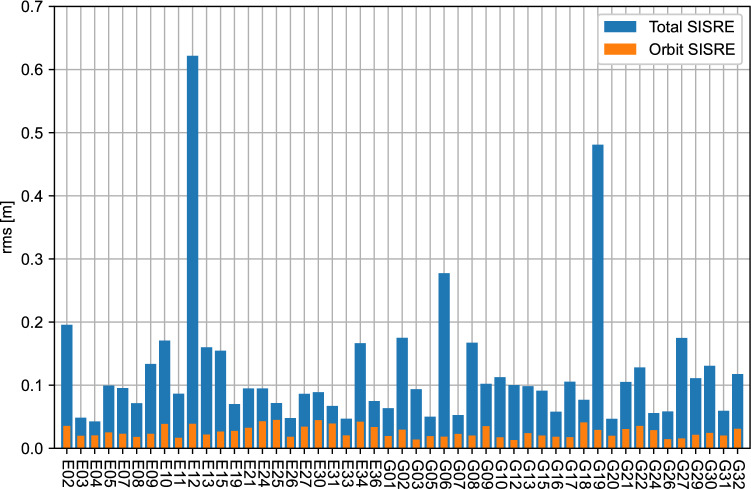
Daily average orbital SISRE (orange) and total SISRE (blue) per Galileo and GPS satellite of HAS corrections relative to final CODE orbits and clocks on day 247 of year 2022

In addition to showing the evolution of SISRE over one day, it is interesting to look at the SISRE per satellite, as presented in Fig. 5. Each bar represents the SISRE rms over the whole day. As expected, all satellites from both constellations have reasonable orbit SISRE at the centimeter level, as well as total SISRE at the decimeter level. The total SISRE is consistent for all satellites, except for G19 and E12 due to their higher clock errors. Similarly to the above results, these SISRE values are comparable to values by Hauschild et al. (2022) and Fernandez-Hernandez et al. (2022), with the exception of the GPS total SISRE, which are improved in the summer 2022 test broadcast.

## Assessment of satellite code bias corrections

In addition to the satellite orbit and clock corrections, the last remaining products to be analyzed from the test campaigns are the code biases. The analysis is performed by comparing the HAS test signal code biases with final Differential Code Biases (DCBs) from the German Aerospace Center DLR (Montenbruck et al. 2014). Given that the DLR DCBs are final products and that they are comparable to the sub-nanosecond level to other DCB products from analysis centers such as CODE and the Chinese Academy of Sciences (CAS), they can be taken as the reference to compare against.

The code bias corrections in the HAS test signal were not computed by the Galileo infrastructure. Instead, they were introduced by configuration based on DCBs from CAS to test and validate the HAS SiS ICD. Therefore, code biases in the test broadcast are not representative of the HAS Initial Service to be declared. As part of the HAS Initial / Full Service provision, code biases will be estimated from the service’s network of reference stations. Nonetheless, the results obtained during the testing campaign are described. The code biases were in the OSB format, meaning that the corrections are pseudo-absolute (“absolute” because each bias is applied directly to its measurement, and “pseudo” because the biases are, in theory, relative, but the user does not need to need to know the reference signals) and can be applied directly to the pseudorange measurements. During the summer 2022, the code bias corrections were broadcast for signals E1 C no data (C1C), E5A Q no data (C5Q), E5B Q no data (C7Q), and E6 C no data (C6C), for Galileo, and L1 C/A (C1C), L2C (C2L) and L2P (C2P) for GPS, with the identifiers in parenthesis being the signal codes in RINEX 3 format.

**Fig. 6:**
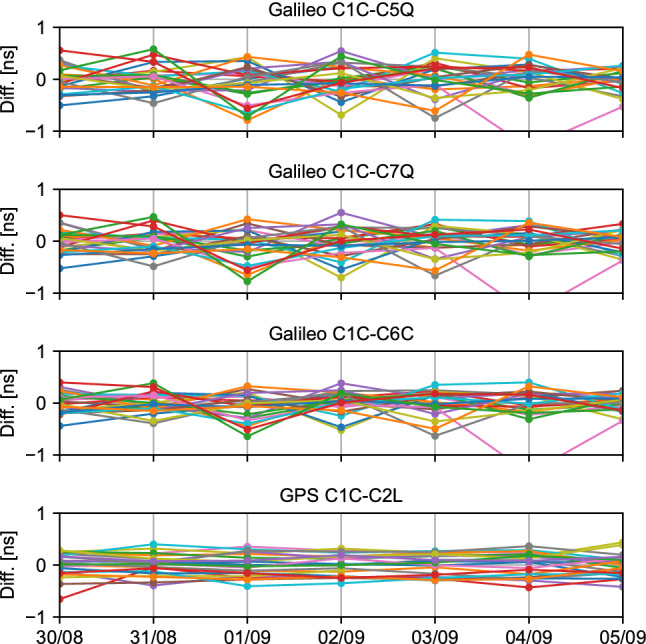
Daily average difference between HAS code biases and final DCB products from DLR. The graphs are for the period between days 242 and 248 of year 2022 for Galileo and GPS satellites/signals. Each color represents a different satellite. The *x*-axis is in the format “day/month”. On September 4th, satellite E25 reaches values of − 1.3 ns, − 1.4 ns, and − 1.4 ns for C1C–C5Q, C1C–C7Q, and C1C–C6C, respectively

In order to perform the comparison with the reference DCBs, the HAS DCBs are created by differencing OSBs. For instance, the HAS C1C–C5Q DCB is recovered by subtracting the C1C OSB from the C5Q OSB. In total, four DCB combinations are analyzed: C1C–C5Q, C1C–C7Q, and C1C–C6C for Galileo, and C1C–C2L for GPS. The C2P bias is not analyzed due to the absence of DCBs involving the signal. The evolution of the DCB differences for all available satellites over one week in summer 2022 is shown in Fig. 6. Each data point represents the average over a day, though the code biases tend to be constant over the course of a day. The code biases in the test campaign are constant with adjustments at 2 am UTC to update the biases. A mean value of − 1.81 ns has been removed from the GPS DCB difference, as the constellation-dependent mean would be absorbed by the receiver clock at the user level.

The figure shows code bias stability over the week, as the code biases from the test campaign are within less than one ns of the DLR DCBs. The difference in DCB between HAS and the DLR products appears to be at the sub-nanosecond-level, with rms of 0.29 ns, 0.27 ns, and 0.23 ns for C1C–C5Q, C1C–C7Q, and C1C–C6C, respectively. For GPS C1C–C2L, the rms is 0.21 ns. These values are in accordance with typical rms values when comparing DCBs generated from different analysis centers (Deng et al. 2021; Li et al. 2017; Villiger et al. 2019). Galileo satellite E25 experiences higher errors on September 4th, as can be seen from all three DCB plots. These higher errors are due to a jump in all HAS biases for that satellite that lasted for 24 h, where the jumps reached 1.7 ns, 3.0 ns, 3.1 ns, and 3.1 ns for C1C, C7Q, C5Q, and C6C, respectively.

**Fig. 7:**
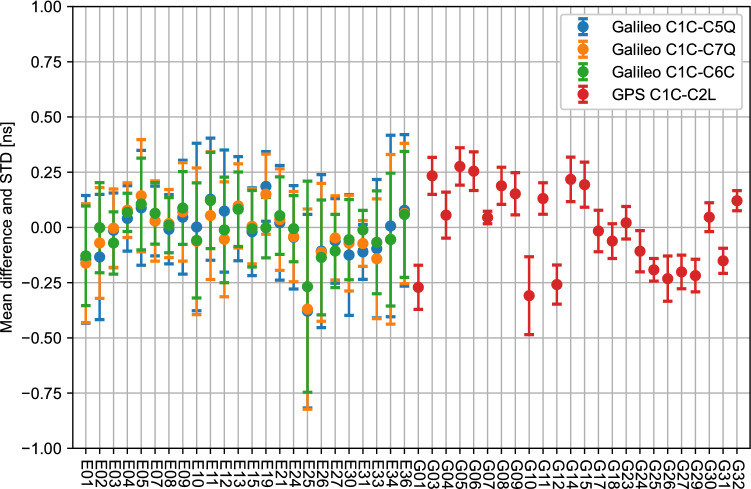
Mean difference and standard deviation per Galileo and GPS satellite of HAS code biases relative to final DCB products from DLR. The statistics are based on one week of data, between days 242 and 248 of year 2022

Figure 7 summarizes the mean and standard deviation of DCB differences for each satellite over the course of the same week for all four DCBs. The figure is intended to show the performance per satellite rather than show the evolution over time, as presented in Fig. 6. Results show good consistency, as most satellites are within 0.50 ns of the DLR DCBs, except for satellite E25 due to its higher errors on September 4th, as shown in Fig. 6. The standard deviation of GPS DCBs is lower than that of Galileo, showing good stability over several days for GPS DCBs. These values show that clock and DCB corrections from HAS are aligned with International GNSS Service (IGS)-type corrections, as clock and DCB differences are at the sub-nanosecond level.

### Precise point positioning performance

The performance of HAS products is analyzed by generating PPP solutions using the HAS corrections. The current section begins with a description of the processing strategy used for the PPP processing, as well as a description of the data being processed. The section continues with PPP results from the summer 2022 test broadcast, before ending with a preliminary analysis of the effect of HAS correction latency on PPP performance.

#### Processing strategies and data

**Fig. 8:**
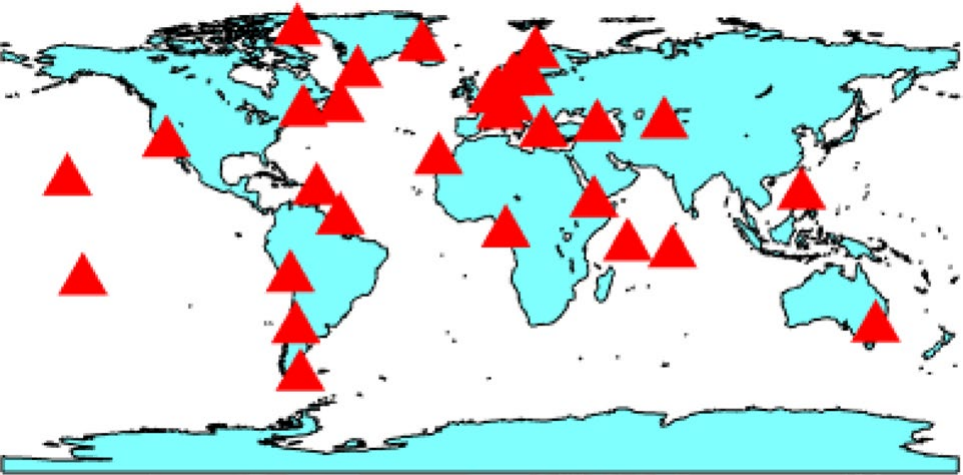
Map of the stations used in the PPP processing and analysis

The PPP processing is performed with the well-tested York-PPP engine from York University’s GNSS Lab. The software has been modified to accept HAS corrections as input, as well as any modifications related to the nature of the corrections, i.e., orbit corrections relating to the ionosphere-free antenna phase center, validity intervals, etc. The PPP tests are performed on a set of 33 global IGS stations. A map of the stations is provided in Fig. 8. Given that HAS GPS code biases are only available for the C1C, C2L, and C2P signals, the stations are selected by ensuring that they track C1C and at least one of the two L2 signals. The tests are performed over a period of one week between days 242 and 248 of year 2022. A total of approximately 1200 independent three-hour long datasets are processed.

The observation data are processed with the processing strategies described in Table 3 using a sequential Least Squares filter. Both Galileo and GPS measurements are processed using HAS orbits, clocks, and code biases. All data are processed in dual-frequency mode (E1/E5b for Galileo, and L1/L2 for GPS) and in uncombined processing (using raw single-frequency measurements without forming linear combinations of measurements). The satellite and receiver antenna corrections are applied from the IGS14 ANTEX file (Schmid et al. 2016). All other necessary corrections are applied, including phase wind-up, relativistic effect, and Earth rotation following the IERS conventions (Kouba and Mireault 1998). An elevation angle cut-off of 7° is applied to reject satellites near the horizon from the processing. To recover satellite orbit and clock corrections from HAS corrections, broadcast orbits and clocks are computed using daily merged navigation files from the IGS.

**Table 3:** PPP processing strategy

Parameter	Processing strategy
Receiver coordinates calculation modes	Kinematic mode: estimated with process noise equivalent to 100 km/h Static mode: estimated as constants
Receiver reference coordinates sources	IGS SINEX positions
Receiver clocks	One clock per constellation, estimated as white noise
Tropospheric delay	Dry component: GMF model and mapping function (Kouba 2009) Wet component: Estimated as a random walk process
Ionospheric delays	Estimated as white noise
Ambiguities	Estimated as constant over each continuous arc
Satellite orbits, clocks, and code biases source	Galileo HAS
Measurement weighting strategy	$\sigma =\frac{{\sigma }_{90}}{a+\left(1-a\right)\mathrm{sin}\lambda }$ with ${\sigma }_{90}$ set to 0.1 m and 0.001 m for code and phase measurements, respectively, based on a residual and measurements quality analysis, and $a$ set to 0.15. $\lambda$ is the satellite’s elevation angle

For the analysis, the horizontal and vertical convergence times are defined as the times it takes to reach and settle below 20 cm and 40 horizontal and vertical accuracy, respectively. These definitions are based on the Galileo target performance metrics (EUSPA 2020). In terms of accuracy, the rms of the position errors is assessed over the whole duration of the processing, including the convergence period.

#### Positioning results

**Fig. 9:**
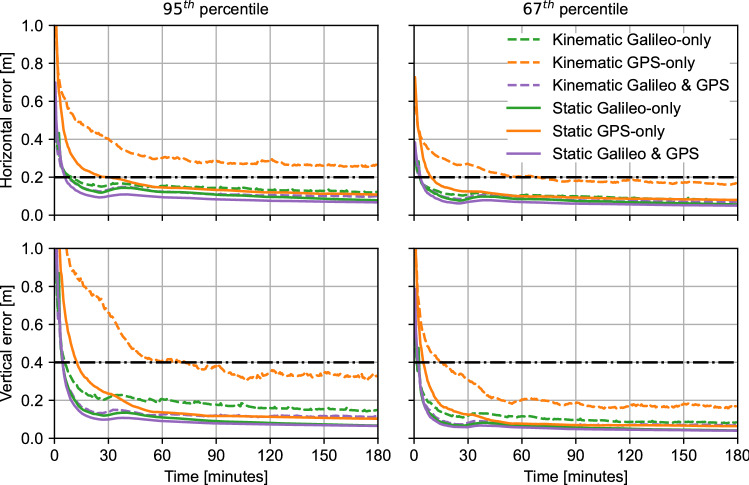
Average PPP horizontal (top row) and vertical (bottom row) performance at the 95th (left column) and 67th (right column) percentiles. The solutions include Galileo-only, GPS-only, and Galileo + GPS HAS results in both static and kinematic modes. Horizontal dashed lines represent target HAS performance

The results from processing the observation data are shown in Fig. 9. The latter illustrates the evolution over time of the average horizontal and vertical errors at the 95th and 67th percentiles for the data being processed in both static and kinematic modes. Each solution in the figure is generated by taking the average of the 95% and 67% lowest position errors on every epoch for each constellation and static/kinematic combination.

The figure shows expected general performance: the solutions start at the few-decimeter level and converge to better accuracies over time; using two constellations provides better performance than a single-constellation solution; and static processing provides better performance than kinematic processing. Additionally, the GPS-only solutions appear to be worse than the Galileo-only solutions. This behavior is due to the GPS solutions tending to have a lower number of processed satellites—mainly due to the limitations in the availability of signals for the code biases, as the average number of processed GPS satellites across all datasets is 5.4, compared to 7.3 for Galileo.

**Fig. 10:**
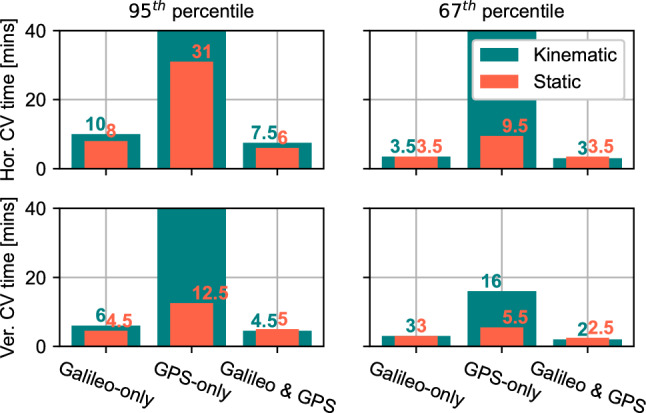
Convergence time statistics for Galileo-only, GPS-only, and Galileo + GPS HAS solutions in both static and kinematic modes. The statistics correspond to the results in Fig. 9

Figure 10 summarizes the convergence time statistics to 20 cm horizontal and 40 cm vertical accuracies for the solutions in Fig. 9. The results show that the convergence time target performance for the HAS full Service of 5 min is met at the 67th percentile level for the Galileo-only and combined Galileo and GPS solutions, and nearly met at the 95th percentile. As expected, the GPS-only results take much longer to converge than the Galileo-only solution, though it is worth noting that adding GPS to the Galileo-only solution improves the latter’s performance. In terms of differences between static and kinematic processing results, other than the GPS-only solution that suffers from less satellites being processed, all other solutions provide comparable results between processing modes. These statistics are reasonable and are expected to improve prior to the transition to SL1. This statement is especially relevant given that the broadcast session was a test broadcast and that the focus was on achieving the targeted accuracies rather than the targeted convergence time. Recall that the HAS specifications consider the use of both Galileo and GPS, with their code and phase biases. The combined Galileo and GPS solution is close to the convergence time target performance for HAS full service with convergence times of 6 min and 7.5 min in static and kinematic modes, respectively.

**Fig. 11:**
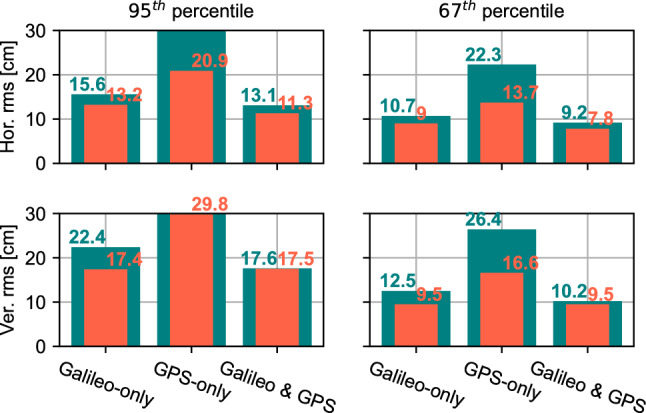
rms statistics for Galileo-only, GPS-only, and Galileo + GPS HAS solutions in both static and kinematic modes. The rms is computed over the whole three hours of processing. Green and red bars represent kinematic and static mode results, respectively. The 95th percentile vertical rms is 30.4 cm and 52.9 cm in static and kinematic modes, respectively

In addition to the convergence time, the solution accuracy is analyzed and summarized in Fig. 11 in the form of the overall rms over the whole three hours. The figure shows that all solutions meet the Galileo HAS specifications of 20 cm horizontal error and 40 cm vertical error at the 95th percentile, apart from the GPS-only solution. Other than that solution, all Galileo-only and combined Galileo and GPS solutions exceed the specifications. Given the HAS full service target performance, the results processed linked to the HAS test corrections meet the user performance specifications in terms of accuracy.

#### Effect of simulated correction latency on HAS position results

In terms of real-time PPP, the performance at the user side depends not only on correction accuracy, but also on the latency of the corrections, which is the difference between the time at which corrections were supposed to be applied and the time they were actually applied. Latency of SSR corrections tends to range from a few seconds to a few tens of seconds (Hadas and Bosy 2015; Wang et al. 2018).

**Fig. 12:**
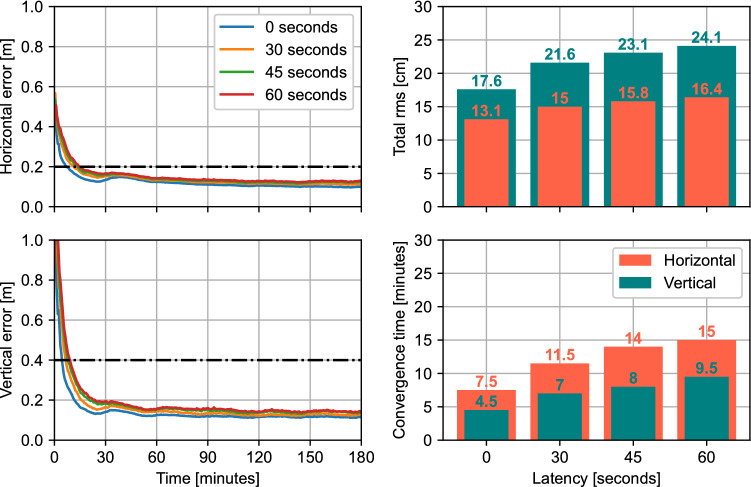
Effect of simulated Galileo HAS correction latency on PPP performance at the 95th percentile based on processing all datasets described in the previous section. The data are processed in kinematic mode using Galileo and GPS satellite orbits, clocks and code biases. The horizontal lines represent the target accuracy performance

Figure 12 shows the effect of Galileo HAS latency on the performance at the 95th percentile from processing all datasets used in the previous figures. The latency is simulated by delaying the application of the HAS corrections by a set number of seconds. The processing is performed in kinematic mode, with both Galileo and GPS corrections being applied. As expected, the solution performance gets worse with higher latencies, as corrections start being applied later than the duration of their validity periods. The performance degradation affects both accuracy and convergence time, as the rms increases from 13.1 and 17.6 cm to 16.4 and 24.1 cm for the horizontal and vertical components, respectively, when comparing the absence of simulated latency to a latency of 60 s. It should be noted though that the HAS target performance of 20 cm horizontal and 40 cm vertical accuracies is still met, even with a latency of 60 s. However, the degradation in convergence time brings the solution even further from the target performance of 5 min convergence, as the convergence time with a latency of 60 s doubles the one without introduced latency. Naturally, these results are only indicators of the scale of the latency’s impact on performance, as the corrections used here are already affected by the real latency of the corrections. Also, service providers apply latency-reducing countermeasures to minimize this effect on position solutions.

## Conclusions and future work

Galileo is the first GNSS constellation to provide a global PPP service to users through its HAS service. With the initial service becoming operational early 2023, the GISCAD-OV project members have had early access to Galileo HAS test signals for experimentation purposes. Live Galileo High Accuracy Service test signals have been broadcast during testing campaigns, where different combinations of corrections have been transmitted and analyzed in an effort to complete an end-to-end experimentation (involving end users) and inject the feedback obtained into the HAS Initial service development final stages prior to entry into Service Level 1. The presented study analyzes HAS test corrections and related performance results based on specific set ups for a summer 2022 testing campaign, where Galileo and GPS satellite orbit, clock, and code bias corrections were broadcast.

This paper shows good availability of corrections, as the average availability is found to be 90.7% for GPS and 96.5% for Galileo over a period of one week. Comparing the broadcasted test HAS corrections to final products from CODE shows good performance. Excluding two satellites for which the clock errors were found to be too large, the rms for the orbit SISRE is 1.6 cm and 1.4 cm for Galileo and GPS, respectively, while the rms of the total SISRE is 10.6 cm and 11.8 cm for Galileo and GPS, respectively, over a period of 24 h. In addition to orbit and clock corrections, satellite code bias corrections were analyzed, and they showed good consistency with final code biases from DLR, with rms of 0.29 ns, 0.27 ns, and 0.23 ns for Galileo C1C–C5Q, C1C–C7Q, and C1C–C6C, respectively, and 0.21 ns for GPS C1C–C2L.

PPP processing was performed on global IGS stations using HAS test signals’ corrections. The results indicate that the HAS accuracy target performance for Full Service of horizontal and vertical rms at the 95th percentile of 20 cm and 40 cm, respectively, was met, as the combined Galileo and GPS solution reached rms of 13.1 cm (11.3 cm) and 17.6 cm (17.5 cm) horizontally and vertically, respectively, in kinematic (and static) mode at the 95th percentile, which are well below the targets. The convergence time target of 5 min was not met, due to the current test campaign not including satellite phase biases, which prevents PPP carrier-phase ambiguity resolution and subsequent improvement in positioning accuracy and reduction in initial convergence time. The combined Galileo and GPS solutions converged below 20 cm horizontal and 40 cm vertical errors within 6 min in static mode and 7.5 min in kinematic mode at the 95th percentile. These results are not considered indicative of the HAS Initial Service depicted in the HAS Information note (EUSPA 2020), although are showing promising results in this specific user scenario. Additionally, simulating latency has shown that a latency of 60 s can still lead to results within the target HAS accuracy performance.

Future work will involve the analysis of campaign results where the whole set of satellite orbit, clock, and code and phase bias corrections will be transmitted for Galileo and GPS, allowing for ambiguity resolution, as well as analysis of multi-frequency processing. Additionally, since the focus of the GISCAD-OV project is cadastral surveying, data that have been collected from surveyors in various locations around Europe will be processed and analyzed.

## Data Availability

The IGS observation data are retrieved from the BKG GNSS Data Center’s server under https://igs.bkg.bund.de/. Galileo HAS corrections analyzed in the current study are not publicly available.
